# Transcriptomic point of departure determination: a comparison of distribution-based and gene set-based approaches

**DOI:** 10.3389/fgene.2024.1374791

**Published:** 2024-05-09

**Authors:** Eduardo Costa, Kamin J. Johnson, Carl A. Walker, Jason M. O’Brien

**Affiliations:** ^1^ Corteva Agriscience, Sao Paulo, Brazil; ^2^ Corteva Agriscience, Indianapolis, IN, United States; ^3^ Ecotoxicology and Wildlife Health Division, Environment and Climate Change Canada, Ottawa, ON, Canada

**Keywords:** toxicogenomics, risk assessment, point of departure, benchmark dose, gene expression, transcriptome, TG-GATEs

## Abstract

A key step in assessing the potential human and environmental health risks of industrial and agricultural chemicals is to determine the toxicity point of departure (POD), which is the highest dose level that causes no adverse effect. Transcriptomic POD (tPOD) values have been suggested to accurately estimate toxicity POD values. One step in the most common approach for tPOD determination involves mapping genes to annotated gene sets, a process that might lead to substantial information loss particularly in species with poor gene annotation. Alternatively, methods that calculate tPOD values directly from the distribution of individual gene POD values omit this mapping step. Using rat transcriptome data for 79 molecules obtained from Open TG-GATEs (Toxicogenomics Project Genomics Assisted Toxicity Evaluation System), the hypothesis was tested that methods based on the distribution of all individual gene POD values will give a similar tPOD value to that obtained via the gene set-based method. Gene set-based tPOD values using four different gene set structures were compared to tPOD values from five different individual gene distribution methods. Results revealed a high tPOD concordance for all methods tested, especially for molecules with at least 300 dose-responsive probesets: for 90% of those molecules, the tPOD values from all methods were within 4-fold of each other. In addition, random gene sets based upon the structure of biological knowledge-derived gene sets produced tPOD values with a median absolute fold change of 1.3–1.4 when compared to the original biological knowledge-derived gene set counterparts, suggesting that little biological information is used in the gene set-based tPOD generation approach. These findings indicate using individual gene distributions to calculate a tPOD is a viable and parsimonious alternative to using gene sets. Importantly, individual gene distribution-based tPOD methods do not require knowledge of biological organization and can be applied to any species including those with poorly annotated gene sets.

## 1 Introduction

Registration of plant protection products (agrochemicals) requires extensive animal and ecological species testing (Sewell et al., 2021). Following current regulatory guidelines, a large battery of toxicity studies is performed to assess toxicity potential across various exposure durations, life stages, and apical endpoints (such as organ histopathology). These data are used to determine the highest dose level producing no adverse effect, which is referred to as the toxicity point of departure (POD) for the molecule being tested (EFSA Scientific Committee et al., 2017; Haber et al., 2018). Currently, benchmark dose (BMD) analysis of apical endpoints is considered the preferred approach for determining a toxicity POD value (Haber et al., 2018). Once the toxicity POD is determined, this value is combined with human exposure or environmental matrix concentration data and uncertainty factors in a risk assessment to identify an acceptable human or environmental exposure level.

Toxicogenomics, the application of genomics to toxicology, has emerged as a potential tool for improving risk assessment (Council, 2007; Mezencev and Subramaniam, 2019). Since molecular changes precede apical effects in a toxicity mode of action (Farr and Dunn, 1999), a comprehensive examination of gene expression change may provide a more efficient and comprehensive approach for identifying exposure levels that are unlikely to induce toxicity (Thomas et al., 2011; Thomas et al., 2013a; Johnson et al., 2022a). Several recent publications have concluded that applying BMD analysis to genome-wide gene expression (i.e., transcriptomics) data from short term exposure studies can derive transcriptomic POD (tPOD) values that are concordant with apical POD values, including apical effects appearing only after long term exposure such as chronic toxicity and cancer (Thomas et al., 2013b; Farmahin et al., 2017; Page-Lariviere et al., 2019; Gwinn et al., 2020; LaRocca et al., 2020; Bianchi et al., 2021).

The most commonly used workflow for deriving a tPOD (Thomas et al., 2007; Yang et al., 2007; Phillips et al., 2019) can be divided into five main steps: 1) input normalized gene expression data (we use the term “gene” generally, but these data may describe different specific endpoints depending on the technology used, such as mapped reads for RNA sequencing, or hybridization probe fluorescence for microarrays); 2) filter genes to keep only those with dose-dependent behavior and a magnitude of change above a defined threshold; 3) fit data for each gene to a dose-response model and identify a BMD value for each gene; 4) map genes with a BMD value to gene sets (e.g., ontologies, pathways, co-expression networks based upon biological knowledge) and identify enriched gene sets; 5) derive a tPOD, the most common method being based on the enriched gene set with the lowest mean or median BMD of the mapped individual gene BMD values. A tool to implement this workflow, BMDExpress, was first introduced in 2007 (Yang et al., 2007). Since then, several other tools have been developed that follow similar workflows, such as the Bayesian Benchmark Dose Modeling System (Shao and Shapiro, 2018), DRomics (Larras et al., 2018), BMDx (Serra et al., 2020), FastBMD (Ewald et al., 2021), and toxicR (Wheeler et al., 2023).

Although many variations of the tPOD derivation pipeline described above are possible, there is considerable evidence that these approaches generally produce comparable tPOD results and that a strong correlation exists between a tPOD and a traditionally-derived toxicity POD (Thomas et al., 2013b; Farmahin et al., 2017; Page-Lariviere et al., 2019; Johnson et al., 2020; LaRocca et al., 2020). One possible, simplified variation of this workflow is to omit the gene set enrichment step (sometimes referred to as functional classification) and determine a tPOD directly from the distribution of individual gene BMD values. Here, these methods will be referred to as distribution-based methods. Farmahin et al. (2017) compared the tPOD values based on eleven different gene-grouping methods, including gene set- and distribution-based approaches, across six different rodent data sets. Regardless of method used, tPOD values were within 10-fold of apical POD values and often less than 3-fold, supporting their conclusion that a variety of approaches can be used to determine a tPOD. Recently, Reardon et al. (2023) analyzed seven tPOD methods, four distribution-based methods and three gene set-based methods for *in vitro* transcriptomic data. Overall, the results demonstrated a high concordance among the different methods.

Here, we tested the hypothesis that distribution-based methods for determining a tPOD will generate similar results as gene set methods, while avoiding potential issues related to the gene set enrichment step, namely: the loss of information imposed by incomplete annotation of genes, and the unreliability of drawing mechanistic conclusions from the gene set driving the tPOD.

We tested our hypothesis by analyzing rat liver transcriptome data from a set of 79 molecules from a public database (Igarashi et al., 2015). Gene set-based results were generated using four different gene sets: 1) gene ontology biological process classifications (GOBP) (Consortium, 2004); 2) BioPlanet pathways (Huang et al., 2019); 3) REACTOME pathways (Jassal et al., 2020); and 4) a gene set derived from a co-expression network built using weighted gene co-expression network analysis (WGCNA) (Sutherland et al., 2018). Transcriptomic POD values derived using these genes sets were compared to tPOD values derived from five distribution-based methods: a method based on the curvature of the accumulation plot of BMD values (Johnson et al., 2022b); a method based on the first mode of the BMD distribution (Page-Lariviere et al., 2019); two percentile methods (fifth and 10th percentile of the gene-specific BMD values) (Farmahin et al., 2017; Reardon et al., 2021; Reardon et al., 2023); and the 25th lowest ranked BMD method (Reardon et al., 2021; Matteo et al., 2023; Reardon et al., 2023). Results support the hypothesis tested and suggest that distribution-based tPOD derivation is a viable and parsimonious alternative to gene set-based methods.

## 2 Materials and methods

### 2.1 Data

Data were extracted from Open Toxicogenomics Project-Genomics Assisted Toxicity Evaluation Systems (TG-GATEs) which is a database created by the Japanese Toxicogenomics Project containing apical toxicity and microarray-based transcriptome data (liver and kidney) generated on 170 molecules (mostly pharmaceuticals) (Igarashi et al., 2015). Liver transcriptome data from the 29-day exposure protocol were used in the present study. Transcriptome data were generated using whole-genome Affymetrix microarrays (Affymetrix Rat 230 2.0).

The 170 molecules in the TG-GATEs database were filtered to a set of 79 molecules with sufficient and appropriate data for benchmark dose (BMD) modeling. This is the same working dataset used in (Johnson et al., 2020). The 79 molecules were chosen according to the following criteria: 1) liver transcriptome data available for more than one treated dose level; 2) apical data collected across all liver endpoints in the study; 3) at least one apical endpoint with a treatment-related effect; and 4) at least one dose level at 29 days of exposure with no or a modest (<20% effect size) effect across all apical endpoints examined. Although analysis of apical endpoints was outside the scope of this work, the apical filtering criteria helped to ensure the appropriateness (at least one dose level with no or little response and one dose level with a robust response) of the dataset across the 79 molecules examined in the present study.

Supplementary File S1 contains gene expression data for the 79 TG-Gates molecules, which have been normalized using the Robust Multi-array Average algorithm (Irizarry et al., 2003) and log-transformed (base 2).

### 2.2 Benchmark dose (BMD) analysis

BMDExpress software (version 2.2; build 0148) was used to perform BMD analysis on the TG-GATEs microarray data (Phillips et al., 2019) according to the National Toxicology Program (NTP) recommendations, which are described in detail elsewhere (NTP, 2018). Briefly, prior to model fitting, normalized microarray probeset data were filtered against a Williams trend test, test *p*-value <0.05, and an absolute fold change >1.5 to identify dose-responsive probesets. For probesets passing this filter, expression data were fit to Hill, power, linear, polynomial 2, exponential 2, exponential 3, exponential 4, and exponential 5 dose-response models. A best fit model for each probeset was selected using the following settings/parameters: 1) maximum iterations of 250; 2) confidence level of 0.95; 3) constant variance; 4) a nested Chi-square test with a *p*-value <0.05 to identify the best polynomial model; 5) power restricted to ≥1; 6) Hill models with a k parameter <1/3 of the lowest positive dose were flagged; when flagged, the next best model with a *p*-value >0.05 was used; 7) lowest Akaike Information Criterion value. The best fit model for each probeset was used to determine a probeset-specific BMD, and its upper and lower 95% confidence limits, BMDU and BMDL, respectively, based on a benchmark response equal to one standard deviation of the control mean (Davis et al., 2011). Probesets with modeled BMDs > the highest dose level or BMDU/BMDL ratios >40 were removed from further analysis.

### 2.3 Gene set-based tPOD determination

Gene set enrichment analysis was performed within BMDExpress on the probesets resulting from the BMD analysis described in Section 2.2. Probesets were mapped to unique genes based on NCBI Entrez Gene identifiers. When two or more probesets were associated with a single gene ID, their BMD values were averaged to obtain a single BMD value associated with the gene. If the same probeset was associated with more than one gene, it was excluded from the analysis. Subsequently, genes with an estimated BMD value were mapped to gene sets as described below (Consortium, 2004). Once gene sets were populated with gene BMD values, gene sets were filtered based on the NTP recommended minimum enrichment criteria (NTP, 2018). Namely, retained gene sets were required to have a minimum of three genes and a minimum of 5% of genes with an estimated gene BMD value. In addition to the NTP recommendation, we tested how different minimum enrichment filtering criteria influenced the ability of the method to calculate a tPOD for a given molecule (see Section 2.7 for more details on evaluation metrics). The eleven filters examined are described in Supplementary Table S1. Filter F1 was the least stringent, and filter F11 was the most stringent. Filter F3 corresponded to the NTP recommended criteria. More stringent filters reduce the number of gene sets that meet the enrichment criteria. If no gene set is enriched, then no tPOD value is generated.

We performed gene set enrichment using four gene set structures: 1) GOBP (Consortium, 2004), version from 07/09/2019; 2) BioPlanet (Huang et al., 2019), version from 06/14/2019; 3) REACTOME (Jassal et al., 2020), version from 11/16/2020; and 4) a gene set structure based on a rat liver weighted gene co-expression network analysis (WGCNA) (Sutherland et al., 2018). The GO consortium defines a biological process as a biological objective (e.g., cell growth) to which genes or gene products contribute (Consortium, 2004). BioPlanet is a collection of approximately 1700 non-redundant pathways operating in human cells which was built from the BioCarta, KEGG, NCI-Nature PID, REACTOME, Science Signaling, and WikiPathways databases. Of these databases, the largest number of pathways that contributed to BioPlanet (1,283 pathways) was from REACTOME. Pathways from the different sources were merged in a non-redundant manner, manually curated, annotated, and grouped into categories. The GOBP ontology was also used in the annotation/grouping process which reduced the number of BioPlanet pathways by approximately 10% (Huang et al., 2019). REACTOME is a manually curated database of human signaling and metabolic molecules which are linked based upon a common biological function (Jassal et al., 2020). WGCNA gene sets were built based upon genes which showed correlated expression behavior in rat liver (co-induced or co-repressed) across the Drug Matrix database (Ganter et al., 2005).

Note that we use the term “gene set structure” to denote a set of gene sets, where a gene set is a set of biologically related genes which are clustered in the gene set structure. For example, GOBP will be referred to as a gene set structure, and each of the biological processes described by GOBP ontology is a gene set.

For the sake of reproducibility of results, the annotation files used for each of the four gene sets are provided in Supplementary File S2. For each gene set structure, two files are provided: one mapping from probesets to genes and one mapping from genes to gene sets. These annotation files can be loaded into BMDExpress when performing the gene set enrichment step using the “Defined Category Analysis” feature.

### 2.4 Gene set enrichment based on randomly generated gene sets

Gene set enrichment was also performed with randomized gene sets based upon the structures of GOBP, BioPlanet, REACTOME, or WGCNA gene sets. Randomized gene set structures were generated by replicating the original structure (number of gene sets and number of genes per gene set) of GOBP, BioPlanet, REACTOME, and WGCNA but with random mapping of genes to gene sets. For every gene set structure, this sampling process was done independently for every gene set (i.e., if one gene was randomly assigned to a gene set, it did not decrease its likelihood to be assigned to other gene sets). This allowed a gene to potentially be assigned to more than one gene set, which reflects the original structures of GOBP, BioPlanet, and REACTOME. However, since the original WGCNA structure had non-overlapping gene sets, we applied an alternative randomization procedure to WGCNA that ensured that each gene appeared only once in the randomized structure.

The randomization process was repeated 1,000 times for each gene set structure. Enrichment analysis using these randomized gene sets was performed in the same way as described in the previous section, using the recommended NTP enrichment criteria filter. If at least half of the random simulations (i.e., 500 simulations) of the gene set enrichment step returned a tPOD value for a given molecule, the median across all simulations resulting in a tPOD value was reported; no value was reported for the molecule otherwise. The fold change between the tPOD value derived from the original gene sets and the median of the tPOD values across simulations was calculated for each molecule. This allowed for standardized comparisons of the effects of random versus the non-random gene sets for GOBP, BioPlanet, REACTOME, and WGCNA.

The randomized gene set enrichment simulation can be performed in BMDExpress by using the “Defined Category Analysis” feature with modified versions of the gene-to-gene sets mapping file provided in Supplementary File S2. The randomization of the gene sets’ content was performed using the sample() function from R version 4.1.2.

### 2.5 Gene set enrichment from artificially down-sampled gene expression data

Gene set-based tPOD determination was performed on artificially down-sampled gene expression data. Namely, a down-sampling procedure was performed using the sample() function from R version 4.1.2 to select only a portion of the original probesets in the expression file; the list of original probesets can be obtained from the expression files in Supplementary File S1. The files in Supplementary File S2 were then modified to keep only the probesets and, consequently, genes present in the down-sampled set. The modified files were then used as input for the “Defined Category Analysis” feature of BMDExpress. This procedure created versions of the original gene set structures where every gene set in the original structure was replaced by a subset of that gene set. In contrast to the procedure used in Section 2.4, where the number of gene sets and gene set sizes were kept the same (but with random content), the procedure described in this section reduced the original gene set structure.

Three different scenarios were used to artificially reduce the original input data. In the first two scenarios, the reduction was random, while in the third scenario it was based on biological knowledge. More specifically, Scenario 1 randomly retained 60% of the original probesets, while Scenario 2 randomly retained 11.3% of the original probesets. Finally, Scenario 3 selected only probesets that map to S1500+ rat genes. The S1500+ is set of approximately 3,000 “sentinel” genes that were selected using both computational and knowledge-driven approaches that can provide broad coverage of pathways deemed toxicologically relevant using only one 10th of the full transcriptome (Mav et al., 2018). The S1500+ was developed as a more economical alternative to measuring the whole transcriptome to reduce costs associated with using genomics for chemical testing. Note that the percentage chosen for Scenario 2 (i.e., 11.3%) approximated the percentage of rat microarray probesets that are mapped to S1500+ rat genes (Scenario 3). The list of probesets used in Scenario 3 is provided in Supplementary File S3. The percentage chosen for Scenario 1 (i.e., 60%) was arbitrarily chosen to represent a moderate reduction in the number of probesets that map to gene sets, similar to what might occur when using a species with relatively low genome annotation.

The random selection in scenarios 1 and 2 was repeated 1,000 times, and only molecules for which at least half of simulations (i.e., 500 simulations) returned a tPOD value were considered in the analysis of the results. For the scenarios based on random reductions, the output tPOD value for each molecule was the median tPOD across all tPOD values from the 1,000 simulations. The recommended NTP minimum enrichment criteria filter was used in all three of these scenarios.

### 2.6 Distribution-based tPOD determination

Distribution-based tPOD determination started with the list of probeset-specific BMD values provided by the BMD analysis described in Section 2.2. Five distribution-based methods were used to determine the tPOD: an Accumulation Plot Maximum Curvature method (PODAcc), a First Mode method (FirstMode), 5th and 10th percentile methods (Perc05 and Perc10), and a 25th lowest rank gene method (Rank25).

The PODAcc method has been previously described (Johnson et al., 2022b). A log10 transformation was applied to the BMD values prior to the analysis. This method defines the tPOD as the point of maximum curvature of an accumulation plot of individual gene BMD or BMDL (BMD/L) values; in this manuscript, only BMD values were used. The point of maximum curvature was calculated using the Kneedle algorithm (Satopaa et al., 2011).

The First Mode method has been described previously (Page-Lariviere et al., 2019). As in the PODAcc method, calculations were performed in a logarithm scale with base 10. Modes and antimodes were identified as local maxima and local minima, respectively, from distribution kernel density estimates generated with the density function in R. The bandwidth argument of the density function was determined by the Sheather and Jones bandwidth selection method (Sheather and Jones, 1991) with a minimum bandwidth set to 0.015 log10 mg/kg/d. Local maxima required a minimum probability density of 5.5% to be considered a mode. Once modes and antimodes were identified, the first mode was deemed the POD.

Previous studies have used percentile-based methods, particularly the fifth percentile method, to estimate the tPOD (Farmahin et al., 2017; Reardon et al., 2021; Reardon et al., 2023). The tPOD was defined as the lower fifth or 10th percentile of the distribution of probe BMD values across molecules.

The 25th lowest ranked method, previously applied in other studies (Reardon et al., 2021; Matteo et al., 2023; Reardon et al., 2023), sorted the probe-sets by their BMD values in ascending order and defined the tPOD as the 25th BMD value. If the BMD distribution had fewer than 25 values, the method did not produce a tPOD.

Unlike the gene set-based methods, which use minimum enrichment gene set criteria, and the Rank25 method, the other four distribution-based methods do not have any pre-defined minimum criteria to determine a tPOD. To avoid tPOD determination on very few BMD values, we chose an arbitrary minimum threshold of at least 25 probesets with a BMD value.

### 2.7 Evaluation metrics

We used fold change metrics and Root Mean Square Difference (RMSD) to assess the concordance of tPOD results from different methods. The employed fold change metrics were: 1) relative fold change; 2) median absolute fold change; and 3) proportion of molecules with a fold change under a certain threshold k, which shows the fraction of molecules that have similar tPOD values from two methods within a specified range (e.g., less than two-fold difference). The relative fold change metric assessed the agreement between methods at the molecule level, while the latter two metrics summarize the agreement across molecules. RMSD is another metric that provides an average estimate of the agreement of values between two methods and compares which methods are more similar to each other. The RMSD between two vectors of values X and Y is given by the following equation, where N is the length of the vectors.


$$\text{RMSD}=\sqrt{\frac{\sum_{k=i}^{N}{\left(X_{k}-Y_{k}\right)}^{2}}{N}}$$

We converted the values to the log scale with base 10 before calculating the RMSD. This was necessary because the dose level values in the TG-GATEs experiments had different orders of magnitude, and we wanted to focus on the relative concordance of methods rather than the absolute difference between them. A lower RMSD value demonstrates a better agreement between two sets of values.

We also evaluated the sensitivity of the methods, which was defined as the ability of the method to calculate a tPOD for a given molecule. Method sensitivity was reported either as the number or the fraction of molecules with a tPOD generated. A decrease in sensitivity indicates that a change in the method or the input data resulted in a tPOD obtained from fewer molecules.

## 3 Results

We evaluated tPOD results for the gene set-based approach (Sections 3.1–3.3) and the distribution-based approach (Section 3.4). We present the main results in this section and provide additional results for comparisons between each possible pairing of the methods in Supplementary File S4. All the results presented in Supplementary File S4 were obtained using the recommended NTP enrichment filtering criteria.

### 3.1 Gene set-based tPOD determination excludes gene expression information and affects the sensitivity of the tPOD

**FIGURE 1:**
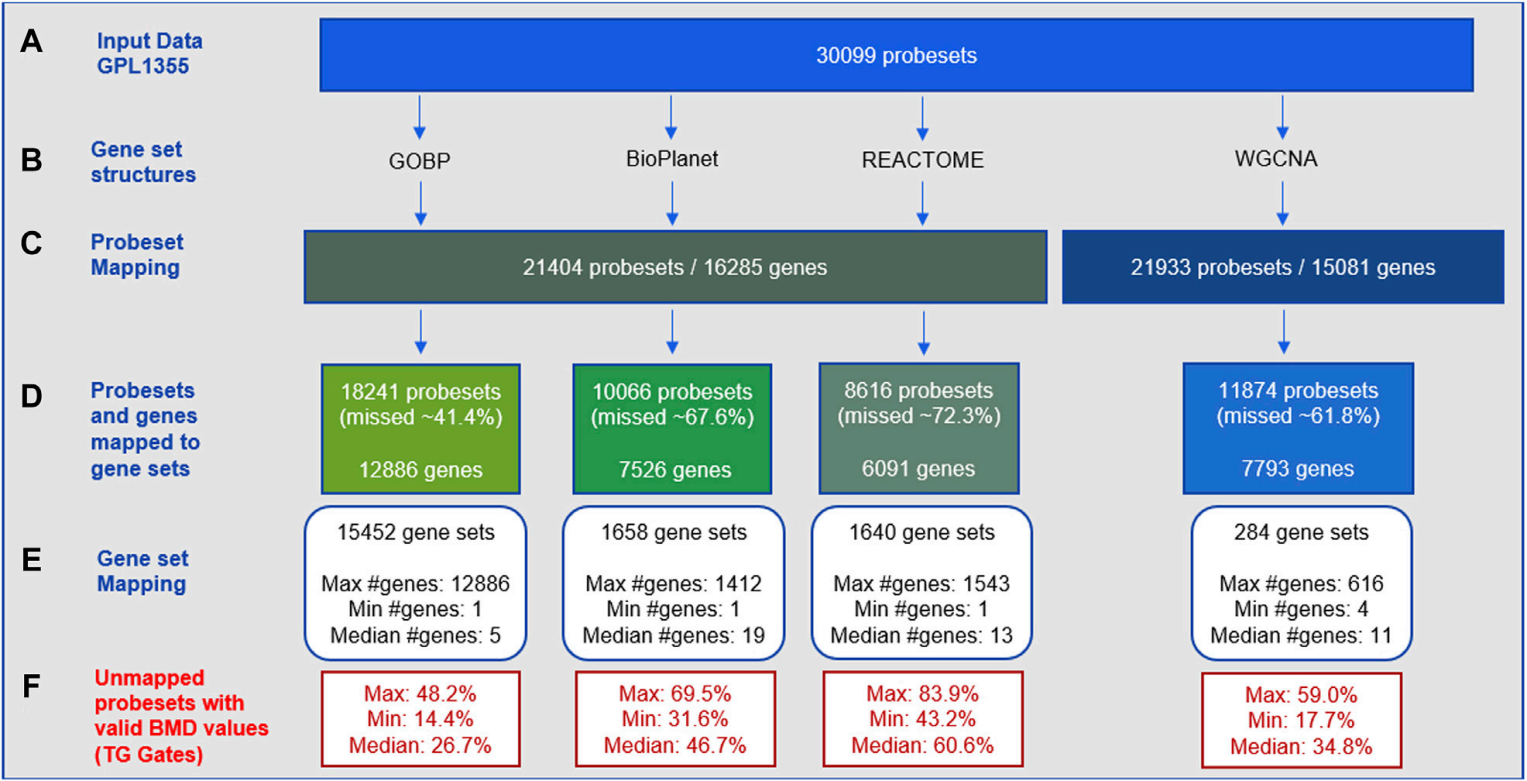
Statistics for probeset mapping to gene set structures for the GPL1355 microarray platform: **(A–E)** experimental-independent probeset mapping; **(F)** experiment-specific reduction of data across 79 TG-Gates molecules. The process of mapping to gene set structures results in the omission of dose-responsive gene expression data.

The gene-set enrichment step often includes only a fraction of the genes in the transcriptome. Figure 1 shows statistics on how the gene set structures used in this study (Figure 1B) removed input gene expression data from the GPL1355 microarray platform (Figure 1A). Figure 1C shows that only a subset of initial probesets were annotated with gene identifiers, and those that were not annotated did not contribute to tPOD determination. The proportion of unmapped probesets for each gene set structure varied from about 41% to 72% of the input probesets (Figure 1D). GOBP retained the most probesets, while REACTOME discarded the most. Note that these omissions are experiment independent. That is, the probesets were excluded because they did not belong to any gene set, regardless of whether these probesets contained information used to determine a tPOD for a molecule or set of molecules.

Among the four gene set structures, GOBP had the highest gene coverage (Figure 1D) and the largest number of gene sets (about 10-fold more than BioPlanet and REACTOME, and 50-fold more than WGCNA) (Figure 1E). The median size of the gene sets was small for all structures (5–19 genes per set) (Figure 1E). The four structures collectively encompassed 15,968 genes of which 2,371 were common to all structures and 4,474 were unique to a single structure (Supplementary Figure S1). GOBP was the most overlapping structure with other gene sets. BioPlanet was the structure that captured the highest proportion of GOBP genes (56%). Supplementary Figure S2 provides additional insights into the architecture of these gene set structures, by displaying the distribution of gene sets based on their sizes. Note that the gene set size is determined by the number of genes within the gene set that are present in the GPL1355 microarray platform. This explains the presence of gene sets of size 1 in Supplementary Figure S2. For all structures, most gene sets have up to 100 genes. BioPlanet, REACTOME, and WGCNA have few gene sets with more than 300 genes: 17, 25, and 6, respectively. GOBP, on the other hand, has 536 gene sets with more than 300 genes.

Figure 1F shows the experiment-specific reduction of data for the 79 TG-GATEs molecules using 29-day rat liver gene expression data. The statistics in the figure summarize the proportion of probesets with a BMD value obtained (i.e., signal from a dose-responsive transcript) that were not used for each gene set structure across the 79 molecules. The average proportion of probesets with a BMD value that was discarded varied from 26% to 60% with GOBP retaining the most BMD values and REACTOME removing the most. Supplementary Figure S3 shows the percentages for each molecule.

**FIGURE 2:**
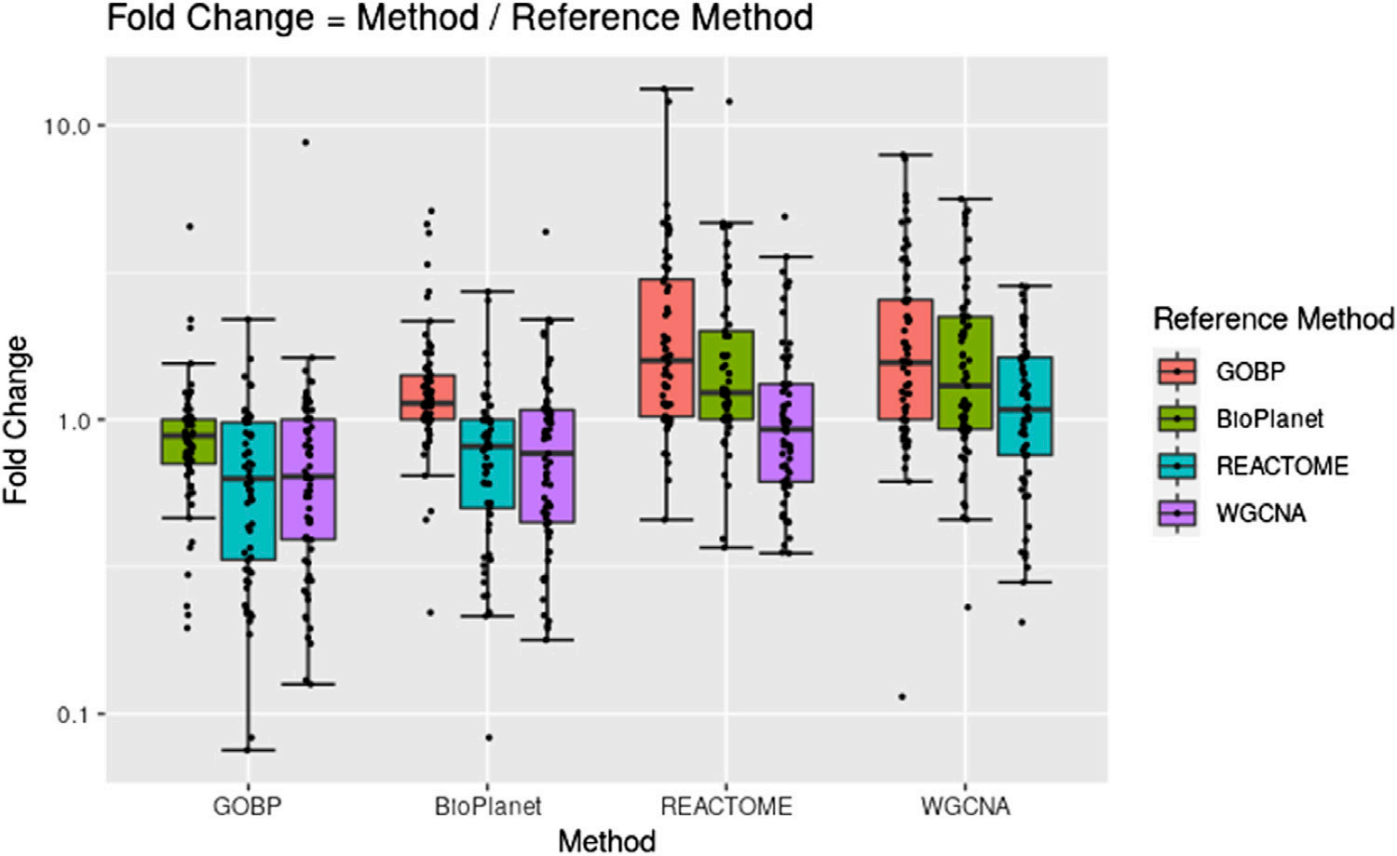
Pairwise comparisons of tPODs for 79 TG-GATEs molecules across four gene set structures using the recommended NTP filter.

Figure 2 shows pairwise comparisons of tPOD values across the four gene set structures for all 79 TG-GATEs molecules using the standard NTP-recommended method (NTP, 2018). The comparisons are shown as fold change ratios of the tPOD obtained for each possible pair of gene set structures. The fold change was calculated by dividing the tPOD of a given gene set structure (labeled on the *X*-axis) by the tPOD of the comparison gene set structure (indicated by the color of the boxplot), which was used as a reference. A fold change of 1 means that the tPOD values were equal. A fold change greater or less than 1 means that the gene set structure in the *x*-axis had a higher or lower tPOD than the structure used as a reference. The results showed that GOBP, which contained the most genes, often produced lower tPOD values than the other three gene set structures. The results were consistent across the four gene set structures (Supplementary File S4): the median absolute fold change ranged from 1.2 to 1.6, the RMSD ranged from 0.22 to 0.39, and at least 61% of the molecules had a tPOD within a factor of two for each pair of structures. The GOBP and BioPlanet structures showed the highest agreement: these had a median absolute fold change of 1.2, a RMSD of 0.22, and 86% of the molecules had a tPOD within a factor of two for each other. The sensitivity of the results (Table 1) was comparable for the GOBP, BioPlanet, and WGCNA structures, which yielded tPOD values for 74, 73, and 72 molecules, respectively. The REACTOME structure, which omitted the most dose-responsive genes, had the lowest sensitivity, returning a tPOD value for only 62 molecules.

**TABLE 1:** Number of molecules with a tPOD value obtained across four gene set structures and for different data strategy reductions.

Ontology	Number of molecules with a tPOD value obtained			
	All microarray data	60% of microarray data	11.3% of microarray data	S1500+
GOBP	74	71	53	73
BioPlanet	73	70	42	73
REACTOME	62	58	31	65
WGCNA	72	67	35	67

**FIGURE 3:**
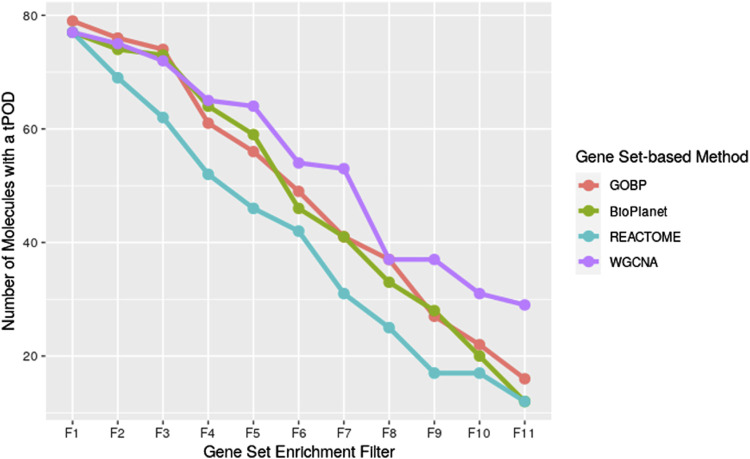
Number of molecules that returned a tPOD across four gene set structures after applying increasingly stringent functional classification filters. F1 is the least stringent filter and F11 is the most stringent. Filter F3 is based on NTP recommendations. The specific parameters of each filter are listed in Supplementary Table S1.

We investigated the effect of both gene set structure and the minimum enrichment criteria on the sensitivity of the results (Figure 3). We applied 11 filters with increasing stringency to the gene sets (see Supplementary Table S1). Filter #1 (F1) had the lowest stringency, and Filter #11 (F11) had the greatest. Filter #3 (F3) used the NTP recommended minimum enrichment criteria. Sensitivity decreased as the filter stringency increased for all structures. Filter F3 had a high sensitivity of about 90% on average. REACTOME had the lowest sensitivity for all filters. WGCNA had the least decrease in sensitivity across filters and was the most sensitive structure for nearly all of the high stringency filters (F4 to F11). The tPOD values for all gene set structures across all 11 filters are available in Supplementary File S5.

To further explore the interaction between the choice of gene set structure and the filtering criteria, Supplementary Figures S4-S7 depict the distribution of gene sets containing BMD values which pass Filter F3 for the tested molecules, for GOBP, BioPlanet, REACOME, and WGCNA, respectively. The figures reveal a bias towards gene sets with sizes ranging from 31 to 100 in passing Filter 3 across the four gene set structures tested.

### 3.2 Biological knowledge-generated and randomly-generated gene sets produce similar tPOD values unless more stringent enrichment criteria are used

To investigate the use of biological information within gene sets for tPOD derivation, a randomization study was performed. The following hypothesis was tested: a tPOD derived from randomly built gene sets will be similar to that derived from biological knowledge-based gene sets. To test the hypothesis, tPOD results from each of the four gene set structures were compared to tPOD values derived from randomized versions of the structures.

**FIGURE 4:**
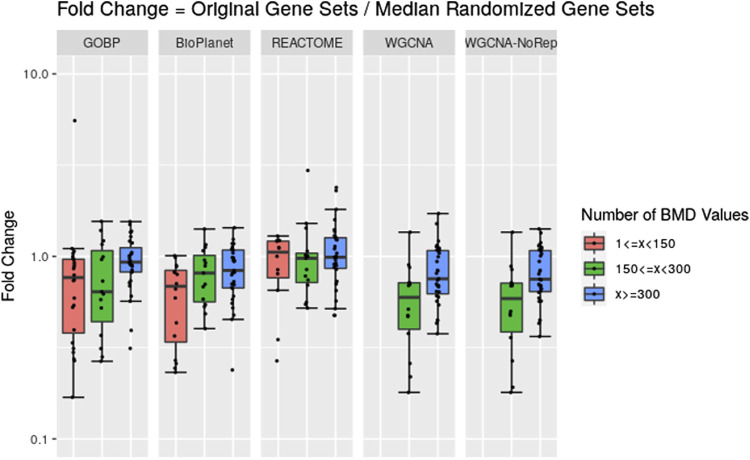
Pairwise comparisons of tPODs derived using the original gene set structure with the median tPOD values from 1,000 randomized versions of that gene set structure. The recommended NTP filter was used for both scenarios. Molecules are split in three groups according to their number of probesets with a BMD value.

Across the molecules studied and using the NTP-recommended minimum enrichment criteria filter, tPOD values obtained from original gene set structures were compared with the median tPOD values from 1,000 randomized versions of the same gene set structure (Figure 4). Results are displayed in terms of fold change, with the tPOD from the original structure in the numerator and the median tPOD from the randomized structure in the denominator. Only molecules for which at least half of the random simulations (i.e., 500 simulations) returned a tPOD value are displayed. The results for each gene set structure were split into three groups according to number of probesets with BMD values obtained used as input to the enrichment step. Out of the 79 tested molecules, 33 molecules belonged to the group with <150 probesets with a BMD value, 15 molecules belonged to the group with ≥150 and <300 probesets with a BMD value, and 31 molecules belonged to with ≥300 probesets with a BMD value. Note that for WGCNA, we used two different randomization procedures: “WGCNA” refers to the same randomization procedure as for the other three structures, where each gene was sampled independently for each gene set. “WGCNA-NoRep” refers to the procedure that ensured non-overlapping randomized gene sets, where each gene appeared only once in the structure. More than half of the randomized WGCNA gene set structures did not yield a tPOD when the number of probesets with a BMD value was less than 150, regardless of the randomization procedure. Consequently, the ratio comparing the tPOD of the original gene set to the randomized genes set structure could not be determined for this group.

The results for GOBP, BioPlanet, and REACTOME showed that when there was a relatively strong gene expression response (more than 300 probesets with a BMD value) tPOD values derived using any of these gene set structures was nearly identical to the tPOD derived using a randomized gene set structure (fold change was close to 1). With the exception of REACTOME, which had similar results for all three response groupings, tPOD values derived for molecules that induced weaker gene expression responses (less than 300 probesets with a BMD value) tended to be lower than the tPOD derived using randomized gene set structures. Overall, we found a high agreement of results between the original and randomized gene set structures when they produced a tPOD value (Supplementary File S4). The median absolute fold change was between 1.3 and 1.4 for all structures. All molecules had a tPOD within a factor of ten for the original and randomized gene set structures, and at least 76% of the molecules had a tPOD within a factor of two. The RMSD was between 0.19 and 0.27 for all structures. For molecules with ≥300 probesets with a BMD value, the results were even more consistent: the median absolute fold change was between 1.2 and 1.4, at least 87% of the molecules had a tPOD within a factor of two, and the RMSD was between 0.16 and 0.19 for all structures.

**FIGURE 5:**
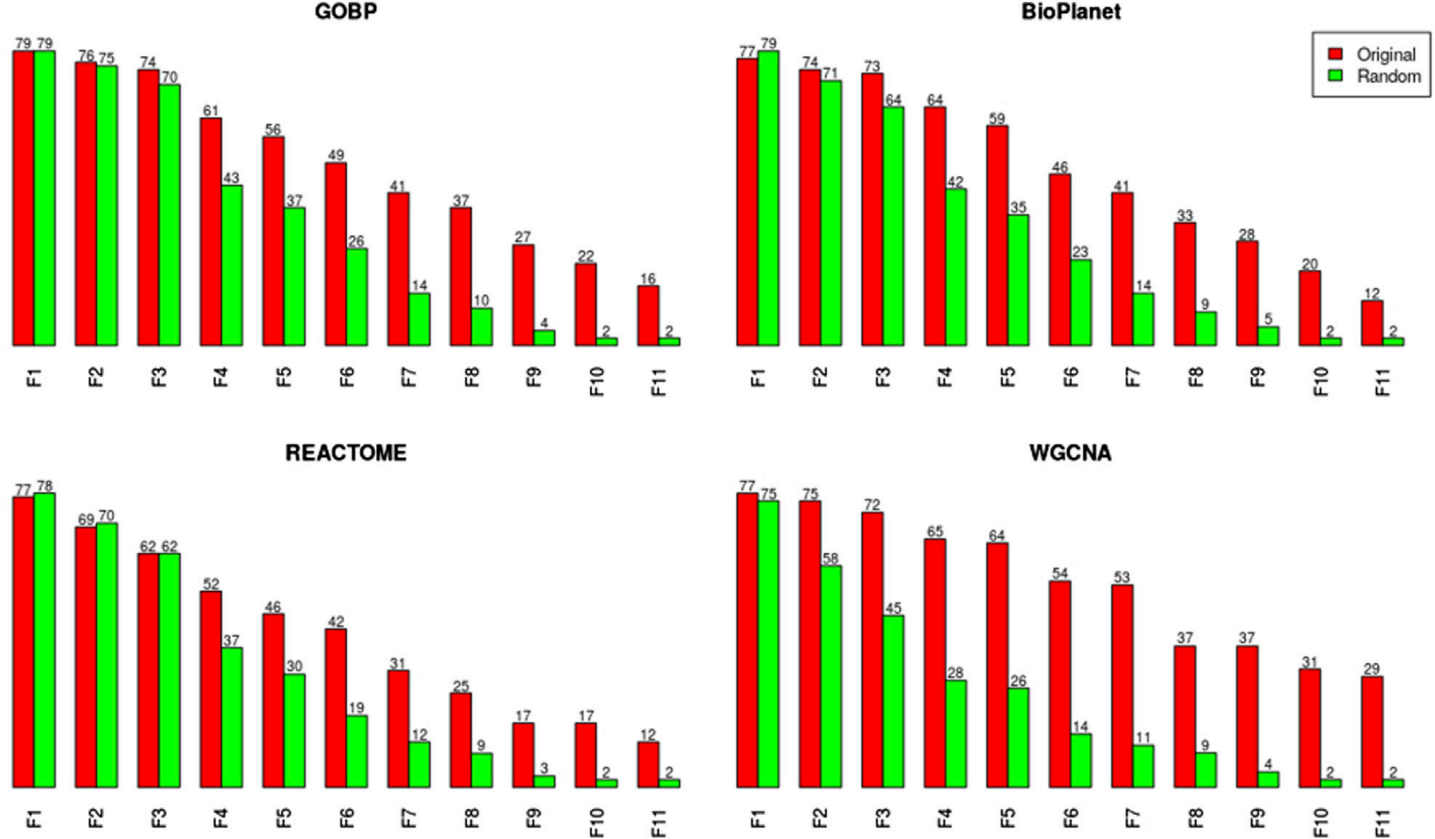
Number of molecules producing a tPOD value for both the original and randomized GOBP gene set structures for increasing stringency of gene set enrichment filtering criteria, as listed in Supplementary Table S1.

We noticed, however, a lower sensitivity for all randomized gene set structures, mainly for molecules with less than 150 dose-responsive probesets. Overall, the sensitivity decreased by 5.4%, 15%, 3.2%, and 37.5% for GOBP, BioPlanet, REACTOME, and WGCNA, respectively. To explore this further, we compared the sensitivity of the original and randomized gene set-derived tPOD values under different enrichment stringency levels. Figure 5 shows how the sensitivity (i.e., the number of molecules with a tPOD) changed for the original and randomized gene set-derived tPOD values when using the different filtering criteria from Supplementary Table S1. We report results only for “WGCNA” and not “WGCNA-NoRep,” because the randomization procedure with non-overlapping gene sets produced identical sensitivity results. The sensitivity of both the original and randomized gene set-derived tPOD values decreased as the filter stringency increased. Except for WGCNA, the original and randomized gene set-derived tPOD values had similar sensitivity when we used the three least stringent filters (filters F1 through F3), including the NTP recommendations (F3). For WGCNA, the sensitivity is similar only for the least stringent filter. For all four gene set structures, the randomized gene sets had a larger drop in sensitivity than the original ones from filter F4 onwards.

The randomization results for GOBP, BioPlanet, REACTOME, WGCNA, and WGCNA-NoRep for each of the 1,000 simulations, under the different filtering criteria from Supplementary Table S1, are available in Supplementary Files S6-S10, respectively. The tPODs calculated from the median across all simulations are reported in Supplementary File S11 for all gene set structures and under different filtering criteria.

### 3.3 Sensitivity is dependent on the number of annotated genes included in the analysis: the effect of poor gene annotation or use of a reduced transcriptome

We hypothesized that the value of the tPOD is dependent on the number of annotated genes included in an analysis. There are several scenarios where the number of annotated genes would be less than ideal. Two specific examples would be when conducting an analysis using a species with a poorly annotated genome, or when using a technology that does not measure the entire transcriptome, such as the S1500+ sentinel genes set (Mav et al., 2018). More specifically, we hypothesized that a tPOD derived using gene set-based methods will generally produce lower tPOD values when using a well-annotated genome or a full transcriptome compared to when using a poorly annotated genome or a reduced transcriptome.

**FIGURE 6:**
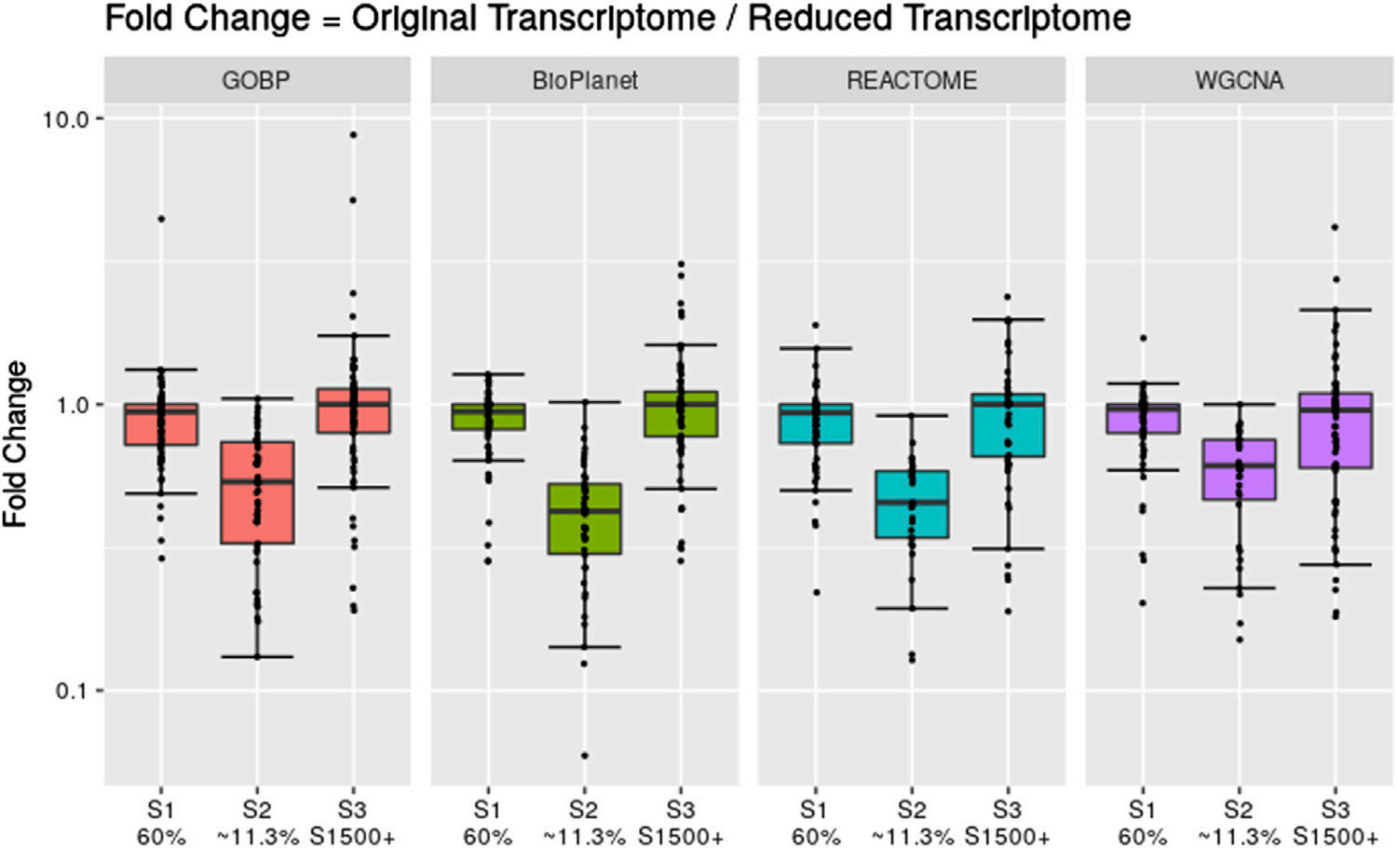
Fold change between the gene set-based tPOD values derived using the full data set compared to tPODs derived using artificially reduced data sets. The number of molecules with a tPOD for each scenario is displayed in Table 1.

To assess the effect of having fewer annotated genes we performed a simulation where we artificially reduced the number of available probesets for the gene set enrichment step. We reduced the data set using three different scenarios: 1) we randomly reduced the number of probesets included in the analysis to 60% of the original data set; 2) we randomly reduced the number of probesets included in the analysis to 11.3% of the original data set; and 3) we only included probesets that are included in the rat S1500+ sentinel gene list. Figure 6 shows the fold change between the tPOD values derived from the original data set with respect to the tPOD values derived from the reduced data set for all four gene set structures under each of the three data set reduction scenarios. Table 1 shows the number of molecules with a tPOD for each structure and scenario.

In Scenario 1, when 60% of the probesets were retained, the estimated tPOD values were similar to those from the original gene set structures (Figure 6; Supplementary File S4), as shown by the small differences in median absolute fold change (1.1–1.2), proportion of molecules within a factor of two (at least 91%), and RMSD (0.15–0.17). This was accompanied by a small decrease (5%–7%) in the number of molecules with a tPOD (Table 1). In Scenario 2, when only ∼11.3% of the probesets were retained, there was a drop in concordance (Figure 6; Supplementary File S4), as shown by the large differences in median absolute fold change (1.6–2.4), smaller proportion of molecules within a factor of two (at least 31%), and larger RMSD values (0.35–0.48). Moreover, there was a significant drop in the number of molecules with a tPOD value (25%–50%) (Table 1). Interestingly, Scenario 3 (S1500+ rat genes) produced tPOD values that were, on average, very close to the tPOD values determined using the original data set (Figure 6; Supplementary File S4), although there was more variance in the ratio when compared to the 60% scenario, as shown by the moderate differences in median absolute fold change (1.2–1.3), proportion of molecules within a factor of two (at least 73%), and RMSD (0.2–0.29). There was also a minimal reduction in the number of molecules with a tPOD value obtained, except for REACTOME, where there was an increase (Table 1).

The results for each of the 1,000 simulations across the different reduction scenarios and gene set structures are available in Supplementary File S12, and the tPODs calculated from the median across all simulations are reported in Supplementary File S13.

### 3.4 Distribution-based and gene-set based PODs are highly comparable

**FIGURE 7:**
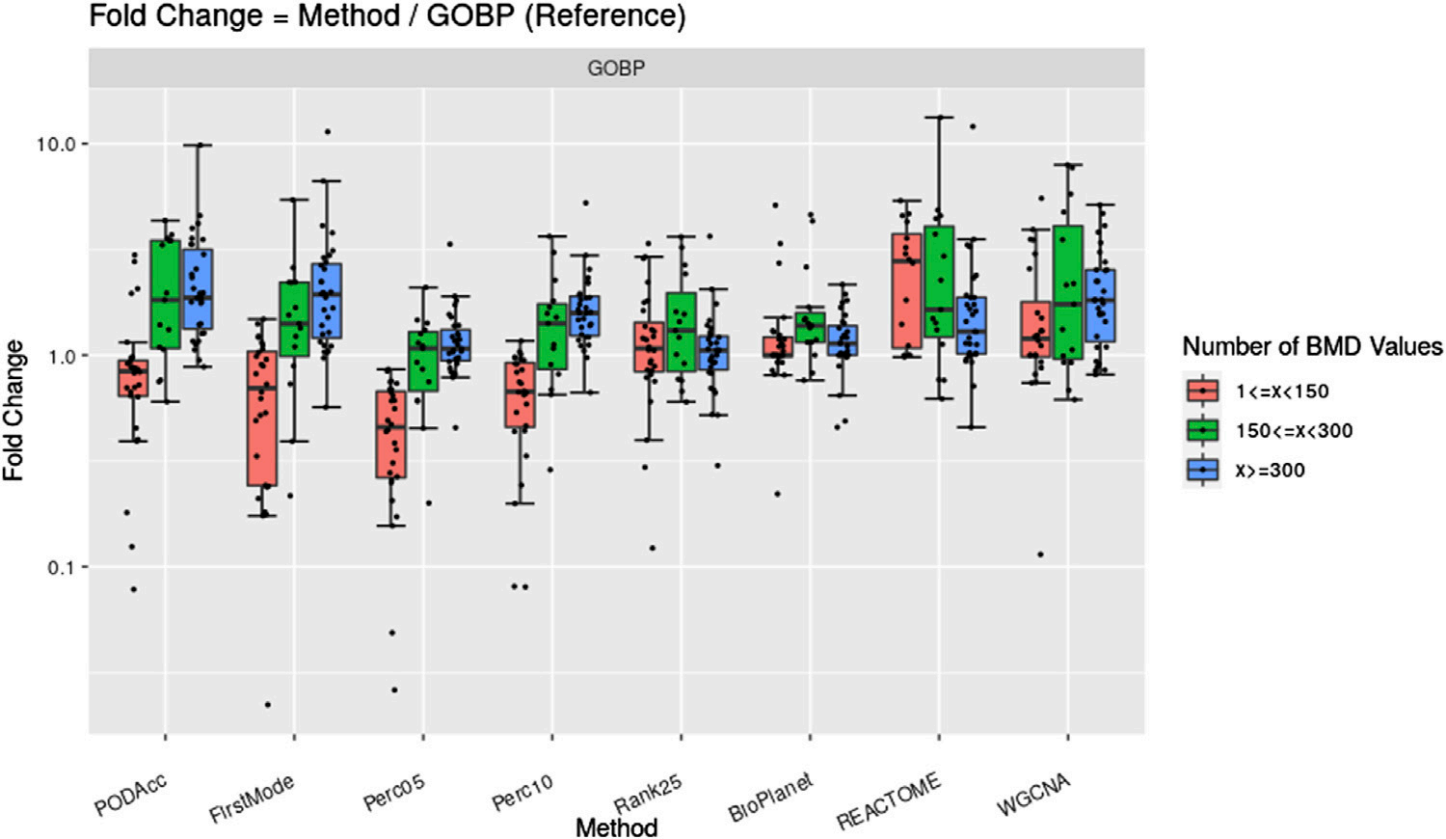
Fold change between tPOD values from five distribution-based methods and the gene set-based method using GOBP. Fold changes for tPOD values from BioPlanet, REACTOME, and WGCNA with respect to GOBP are also displayed. Molecules are split in three groups according to their number of probesets with a BMD value.

Figure 7 shows the fold change difference of gene set derived tPOD values using the GOBP gene set structure and the NTP filters when compared to tPOD values from five distribution-based methods: 1) Accumulation Plot Maximum Curvature method; 2) First Mode method; 3) the fifth percentile; 4) the 10th percentile; and 5) the 25th position gene/probeset. We grouped the results across the 79 tested molecules by the number of probesets with a BMD value obtained that we used as input for the tPOD derivation method. As a reference, we also included in the figure the comparison of tPOD values from GOBP with those from the other three gene set structures.

Methods based on BMD distributions gave similar tPOD values to those from GOBP enrichment (Figure 7; Supplementary File S4). This was more evident for molecules with at least 150 probesets with a BMD value. For these molecules, the distribution-based methods that were most consistent with GOBP were Perc05 and Rank25: median absolute fold change of 1.2–1.3, at least 85% of the molecules with a tPOD within two-fold of one another, an a RMSD between 0.19 and 0.22. Except for FirstMode, all distribution-based methods produced more concordant results with those from GOBP, than when GOBP results were compared with those from REACTOME and WGCNA. Perc05 matched GOBP better than BioPlanet did. When molecules with at least 300 probesets with a BMD value were considered, we observed a very high tPOD concordance for all nine methods tested: for 90% of those molecules, the tPOD values from all methods were within four-fold of each other. When all molecules are considered, regardless of the number of probesets with a BMD value, Rank25 was the distribution-base method which produced the most concordance with GOBP tPOD values: these two methods had a median absolute fold change of 1.3, a RMSD of 0.25, and 81% of the molecules had a tPOD within a factor of two. These results are very close to the concordance between GOBP and BioPlanet, as described previously in Section 3.1. Out of 79 molecules, the distribution-based methods could not determine a tPOD for only one molecule, which had less than 25 probesets with a BMD value.

**FIGURE 8:**
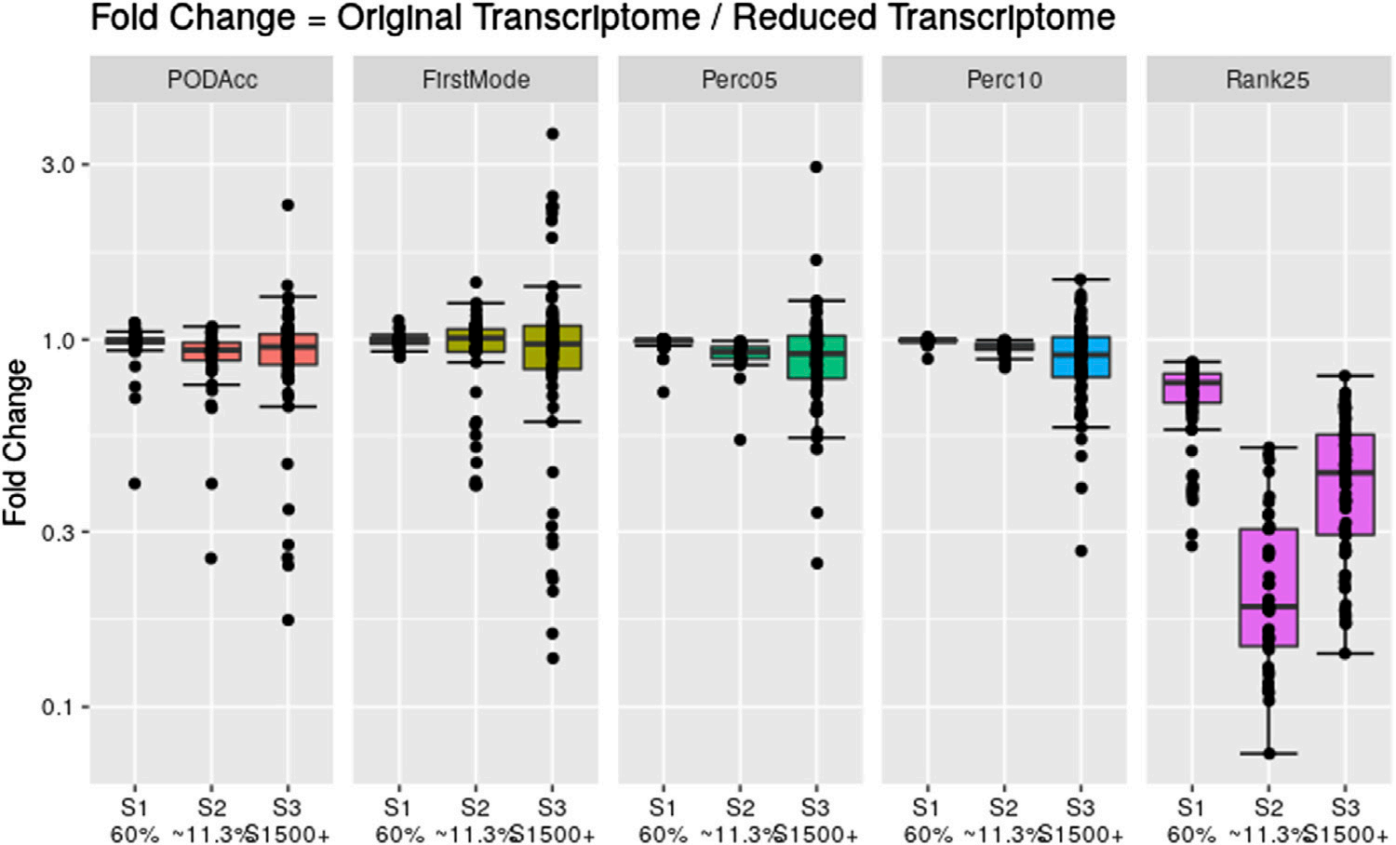
Fold change between the distribution-based tPOD values derived from the original transcriptomes with respect to the ones derived from the reduced transcriptomes. The number of molecules with a tPOD for S1, S2, and S3 is 76, 41, and 67, respectively.

To assess the robustness of the distribution-based methods on reduced datasets, we applied these methods to the same reduced transcriptome datasets used in Section 3.3. Figure 8 shows the fold change difference between the tPOD values derived from the original data set with respect to the tPOD values derived from the reduced data set for all five distribution-based methods under each of the three data set reduction scenarios: S1 (60% of the probesets were randomly retained); S2 (∼11,3% of the probesets were randomly retained); and S3 (only probesets corresponding to S1500+ gene list were retained). Overall, the results for the reduced datasets were very similar to those from the original datasets for the three scenarios tested (Figure 8; Supplementary File S4). More specifically, with the exception of the Rank25 method, tPOD values were nearly identical for S1: median absolute fold change (1.00–1.02), proportion of molecules within a factor of two (at least 99%), and RMSD (0.01–0.06). With the exception of Rank25, tPOD values were also very similar for S2: median absolute fold change (1.04–1.08), proportion of molecules within a factor of two (at least 90%), and RMSD (0.03–0.15). S3 was the scenario with the largest variability in the fold change results: median absolute fold change (1.08–1.17), proportion of molecules within a factor of two (at least 76%), and RMSD (0.14–0.28). The Rank25 method consistently produced larger tPOD values for the reduced transcriptome datasets. Among the three Rank25 scenarios, S1 was the one with least differences in tPOD values, while S2 was the scenario with the most differences. The statistics for the three scenarios, respectively, are as follows: median absolute fold change (1.3, 5.3, and 2.3), proportion of molecules within a factor of two (87%, 2%, and 36%), and RMSD (0.20, 0.72, and 0.45). In terms of sensitivity, the number of molecules with a tPOD returned by the distribution-based methods for S1, S2, and S3 was 76, 41, and 67, respectively.

The distribution-based tPOD results for the original datasets are available in Supplementary File S14. The results for each of the 1,000 simulations across the different reduction scenarios and distribution-based methods are available in Supplementary File S15, and the tPODs calculated from the median across all simulations are reported in Supplementary File S16.

## 4 Discussion

The standard workflow for transcriptomic point of departure (tPOD) determination is composed of numerous choices of parameters and data filtering criteria that might affect the sensitivity of the method and the magnitude of the tPOD value. This work focused on the steps after calculating the benchmark dose (BMD) for each gene (or probeset, since we used microarray data). These steps summarize the BMD values of all genes into a single transcriptome-wide POD value. Gene set enrichment is the most commonly used approach for this aggregation, which consists of mapping genes to gene sets, before making the tPOD call. We investigated several of the key factors related to this analysis, such as gene set structure selection and minimum gene set enrichment criteria. We also compared the tPOD values from gene set enrichment with tPOD values from five distribution-based methods, which bypass the enrichment step and determine a tPOD directly from the distribution of all individual gene BMD values.

One important choice in the gene set enrichment analysis approach is which gene set structure to use. The mapping of expression data to gene sets inevitably leads to data loss, since the gene sets often cover only a fraction of the genes expressed in the transcriptome. For the four gene set structures considered in this study, the median data loss of probesets with a BMD value ranged between 26% and 60% (Figure 1). Data loss could affect the method sensitivity when certain molecules fail to meet the minimum enrichment criteria. Additionally, when fewer BMD values are populated into gene sets, fewer gene sets will have sufficient BMD values to report a median, and thus the lowest median BMD across gene sets (i.e., the tPOD) would be expected to increase. The comparison between the results derived by GOBP and REACTOME illustrates both effects (see Figures 2, 3). GOBP, which is the gene set structure that loses the least amount of data, produced lower BMD values than the other three gene set structures (Figure 2) for most of the molecules and was able to identify a tPOD value for the largest number of molecules when using the recommended NTP filter (F3 in Figure 3). REACTOME, on the other hand, is the gene set structure that removes the largest amount of data, which is reflected in the smallest number of molecules with a tPOD value. Interestingly, WGCNA did not follow this trend. Despite omitting the second lowest number of probesets, WGCNA had similar tPOD values to REACTOME, which excluded the most probesets. This can be explained by the structure of the gene sets. The GOBP, BioPlanet, and REACTOME structures allow the same gene to appear in multiple gene sets and in different combinations with other genes. Mathematically, this will increase the likelihood of grouping genes with BMD values in the same set. The opposite effect of this can be seen in the results based on WGCNA. With a much smaller number of gene sets than the other gene set structures, since WGCNA gene sets are non-overlapping, tPOD results driven by this structure were often larger than for BioPlanet (Figure 2), which has a much larger number of gene sets (Figure 1).

On top of the choice of the gene set structure, the gene set enrichment step also requires the selection of minimum enrichment criteria filters that are used to determine which gene sets can be considered in the calculation of the tPOD. While this filtering process is intended to reduce noise in the tPOD determination, it imposes an additional layer of data omission to the workflow. The stricter the filters are, the less sensitive the method becomes (Figure 3). On the other hand, too relaxed filters might lead to false positive calls. Even though there is a recommendation for the use of relatively relaxed filters by a NTP Toxicology Program Expert Panel (NTP, 2018) and a recent publication found a set of filters that drove tPOD values which were the most concordant with TG-GATEs apical POD values (Johnson et al., 2020), an in-depth empirical discussion about the minimum enrichment criteria filters that takes both sensitivity and false-positive rate into account is still lacking in the field.

Interestingly, WGCNA seemed to be more resistant to the decrease in sensitivity as filter stringency increased, compared to the other gene set structures. WGCNA detected the greatest number of tPOD values for almost all of the filters that were more stringent than NTP recommendations (Filters F4 and greater, Figure 3). One possible explanation for this observation is that genes in the same WGCNA gene sets were found to co-express in rat liver data from the Drug Matrix database. As we used those gene sets in the context of deriving a rat liver tPOD, it was expected that WGCNA would be more likely to group genes with BMD values in the same gene set than the other gene set structures. However, differences in sensitivity between WGCNA and GOBP only appeared for more stringent filters. Investigating the role of co-expressed genes in the sensitivity of the gene set-based approach is, however, outside of the scope of this manuscript and might be an interesting future direction of research. Moreover, it would be interesting to investigate how well WGCNA performs to derive a non-liver tPOD compared to the other gene set structures.

Despite the inherent complexities of the gene set-based methods, one common argument in favor of using this approach is that it provides insight into the biological mechanism behind the transcriptomic dose-response, since gene set structures, such as those used in this manuscript, usually provide a functional annotation for the gene sets. However, even though the gene set structures were built based on biological knowledge, results from our randomization experiment suggest that the most sensitive enriched gene sets, on which the tPOD determination is based, may not be more meaningful than randomly generated gene sets (Figure 4). This was especially prominent when there were greater than 300 probesets with a BMD value (Figure 4) and when relaxed minimum enrichment criteria filters are used, such as the recommended NTP filter (Filter F3 in Figure 5). Interestingly, a comparison between results from Figures 2, 4, which use the NTP filter, reveals that, in general, the randomized versions of GOBP produced POD values that were closer to those given by the original GOBP than when the latter is compared to REACTOME and WGCNA. However, when more stringent enrichment criteria filters were used (Figure 5, Filters F4 to F11), results from the randomized versions of all structures were less sensitive than their original versions. This suggests that the gene sets built upon biological knowledge only have a sensitivity advantage over the random gene sets when enrichment criteria that are more stringent than those recommended by the NTP recommendations are applied. An explanation may be that non-random gene sets are more likely to have more genes with a BMD value than random gene sets and, therefore, are less likely to be discarded by the filtering criteria in the gene set enrichment step. However, for commonly used filters, such as the NTP filter, the results and the sensitivity of the methods were similar, suggesting that the most sensitive non-random gene set is not guaranteed to have any more biological meaning than a randomly generated gene set. This similarity to random gene sets, together with the omission of data discussed in the last paragraph, make gene set-based methods more similar to randomly sampling from a distribution than they might seem at first glance but also have a risk of leading to incorrect biological interpretations. Future research should further investigate how gene set enrichment criteria impact the resulting tPOD values and the associated biological interpretation.

The functional enrichment step in the tPOD analysis requires biologically meaningful gene sets, which may not be available for poorly annotated species. If no suitable gene set structure exists for the species of interest, the gene set-based method cannot be performed. Moreover, the sensitivity of the method may be compromised by a gene set structure with less information (lower gene coverage, fewer and smaller gene sets), as shown by the simulations of reduced gene coverage. When 60% of the original probesets were randomly selected, this effect was minimal. This reduction impacted mainly the molecules with few probesets with a BMD value, resulting in a reduction of 4%–7% in the sensitivity of the gene set methods. However, when only 11.3% of the original probesets were randomly selected, the effect was significant (Table 1). The decrease in sensitivity when we used lower annotation or reduced transcriptomes is explained by the omission of probesets having a BMD value from the gene set enrichment analysis. As a result, in some situations, no gene set passed the enrichment criteria, and the method could not calculate a tPOD for the molecule. For other cases, fewer gene sets met the minimum enrichment criteria, resulting in a general increase of tPOD values (Figure 6). It should be noted that these down-sampling experiments are crude simulations of a poorly annotated genome. A 40% reduction in probes does not necessarily result in a 40% reduction of mapped BMDs. Nevertheless, our results suggest that the number of mapped probes, which is dependent on the degree of genome annotation, can affect tPOD sensitivity and magnitude, although it appears to be most notable when there is a very low number of mapped genes (i.e., when we reduced our probe sets to 11.3%). A more thorough investigation of this effect would be required to quantify the relationship between gene annotation and reduced tPOD sensitivity.

Conversely, reducing the transcriptome by using only probesets mapped to S1500+ rat genes, which are well-annotated and characterized, yielded similar results to the original structure in terms of the tPOD values (Figure 6) and the number of molecules having a tPOD value (Table 1). This may be because the S1500+ gene set contains genes more likely to be affected by toxicant exposure in rat liver, and the remaining gene sets were more likely to pass the percentage criterion of the gene set enrichment analysis. These results indicate that the sensitivity loss from using fewer annotated genes can be alleviated if the retained genes are carefully selected, but this is only possible for species with very well annotated and characterized transcriptomes (which is why S1500+ gene sets are currently only available for human, mouse, rat and zebrafish). These findings suggest that the functional enrichment-based POD workflow is not well-adapted to poorly annotated species.

Overall, our results suggest that gene set derived tPOD values appeared to be sensitive to changes in filtering criteria, gene annotation and/or coverage, and may not provide reliable mechanistic information (based on our randomization experiments), especially when using relaxed filtering. We therefore hypothesized that forgoing the gene set enrichment step, and deriving a tPOD based on the distribution of the gene-specific BMD values would provide comparable results to gene set-based methods, while avoiding some of the shortcomings identified above. As hypothesized, we found that distribution-based tPOD values were highly correlated to gene set tPOD values, especially for compounds eliciting strong gene expression responses (Figure 7). A similar concordance has been reported in previous studies (Farmahin et al., 2017; Page-Lariviere et al., 2019; Reardon et al., 2023).

Furthermore, our results showed that distribution-based methods are more robust to reduced datasets for all the scenarios tested, including random and biology-driven reductions of the transcriptome. With the exception of the Rank25 method, the distribution-based methods from the reduced datasets tend to be very similar to those from the original datasets. As the reduced data is drawn from the original distribution, it has minimum effect on the percentiles, curvature of the accumulation plot, and mode of the distribution. However, for the Rank25 method, a data reduction implied an increase in the estimated tPOD values. This is because this method is based on the BMD value of a fixed position of the sorted list of BMD values. The magnitude of the increase in the tPOD for this method depends on the amount of the reduction of the distribution of BMD values. The larger effect was observed for Scenario 2, where only ∼11.3% of the original probesets were retained. Although Scenario 3 had an equal number of kept probesets, there was a smaller decrease in the list of probesets with a BMD. This is because these probesets were more likely to have a BMD value generated since they were selected based on the S1500+ gene list.

By not requiring the mapping between genes and gene sets, distribution-based methods offer a few advantages over gene set-based methods. First, they simplify the process of determining a tPOD by eliminating the need for the user to define the gene set structure and filtering criteria. Second, they use all the BMD data available, in contrast to gene set-based methods, which only use BMD values for genes/probes that can be mapped to gene sets (Figure 1). Third, the application of distribution-based methods is independent of the biological knowledge related to the species. The method can theoretically be applied in virtually any species, regardless of genome annotation status, which is in contrast to gene set-based methods that require well-annotated ontologies to work well. Finally, the resulting tPOD is “mechanism agnostic.” This last property has one obvious disadvantage: the mechanism of action of the tested compound cannot be inferred directly from this method (although mechanistic investigations can be conducted in parallel). This also comes with two important advantages. First, it avoids biological misinterpretation of the results. As implied by our randomization experiment, there appears to be a risk of incorrectly identifying the mechanism of action when inferred from a gene-set derived tPOD, especially when low stringency enrichment criteria are used. Second, in our opinion, a distribution-based tPOD can be interpreted as the dose level below which a concerted transcriptional change is not expected. This is in principle similar to a NOEL (No Observed Effect Level) and would in theory be protective of all possible adverse effects in the tissue being investigated. This interpretation is in line with the current vision of using a tPOD as a health-protective alternative to a traditional apical endpoint-based POD for chemical risk assessment (Johnson et al., 2022a). In the present study we did not compare how the gene-set and distribution-based tPODs compared to traditionally derived PODs and, therefore, cannot make objective observations about which is more accurate at predicting dose levels associated with adverse apical effects. However, a comparison of gene-set based tPODs to traditional apical PODs was previously conducted for the TG-Gates data set (Johnson et al., 2020) and found a strong correlation (Pearson R = 0.86). Given that we found the distribution-based tPODs to have comparable results to the gene-set based methods (Figure 7; Supplementary File S4), it is likely that these will also be comparable to traditional apical PODs. An evaluation of the various tPOD methods across many diverse data sets might help determine which method provides the most accurate or protective estimates of toxicity POD values.

A caveat of the distribution-based methods is that these may be more prone to false positive results if no minimum criteria in terms of gene expression response are defined. While gene set-based methods will not output a tPOD for molecules with a weak gene expression response if no gene sets meet the minimum enrichment criteria, the distribution-based methods can in theory generate a tPOD even if only a few gene BMD values are obtained. In this work, we used the arbitrary minimum threshold of at least 25 genes with a BMD value. Establishing a minimum gene expression response that must be met in order to apply distribution-based methods is an important research direction. Our data suggest that for transcriptome-wide analyses, this minimum response may be approximately 150 genes with a BMD value.

We acknowledge some limitations of the TG-GATEs data that may affect our analysis and interpretation. One limitation is that the study design of the TG-GATEs data may not be optimal for all molecules, as 33 out of 79 molecules had fewer than 150 probesets with a BMD value. Another limitation is that the TG-GATEs data is based on microarray technology, which may have some disadvantages compared to newer technologies (e.g., RNA-Seq), such as lower sensitivity and data quality issues (Fathallah-Shaykh, 2005; Jaksik et al., 2015). Therefore, we suggest that future studies should use more comprehensive and reliable transcriptomic platforms and designs to improve the accuracy and robustness of the tPOD analyses.

## 5 Conclusion

We investigated two different approaches to summarize gene-specific BMD values into a transcriptome-wide POD: the gene-set enrichment-based approach and the distribution-based approach. We highlighted three main caveats related to the gene set-based methods: 1) the loss of information imposed when mapping to gene sets; 2) the risk of drawing incorrect mechanistic conclusions from the gene set driving the tPOD; and 3) the reduced sensitivity of the gene set-based method when applied to species with poorly annotated genomes. We showed that distribution-based methods produce comparable results to those from gene-set based methods, while avoiding the caveats above. However, several outstanding issues remain regarding best practices for distribution-based methods, such as the minimum gene expression response required to reduce the risk of false positives. Based on these findings, we recommend using a consensus strategy including both gene-set enrichment-based and distribution-based approaches to analyze transcriptomic dose-response data until we can improve the reliability of mechanistic interpretations and understand the behavior of different methods better. This way, we can avoid depending on a single method that may have flaws or biases. Moreover, we advise being careful when drawing mechanistic interpretations from gene-set enrichment-based results. Finally, we recommend the use of distribution-based methods for scenarios with limited biological information and genome annotation coverage.

## Data Availability

The original contributions presented in the study are included in the article/Supplementary Material, further inquiries can be directed to the corresponding author.

## Supplementary material

SUPPLEMENTARY FIGURE S1 Venn diagram illustrating set relation among the gene set structures used in this research.

SUPPLEMENTARY FIGURE S2 Distribution of the number of gene sets based on their sizes for each of the four gene set structures.

SUPPLEMENTARY FIGURE S3 The average data loss of probesets with a BMD value for 79 TG-GATEs across four gene set structures.

SUPPLEMENTARY FIGURE S4 Distribution of GOBP gene sets containing BMD values which pass Filter F3 for the tested molecules: **(A)**: experiment-independent distribution of gene sets based on their sizes, serving as a reference to interpret the other three experiment-specific plots; **(B)**: distribution of gene sets passing Filter F3 for all molecules combined; **(C)**: number of gene sets passing Filter F3 for each molecule across different gene set sizes; **(D)**: percentage of gene sets passing Filter F3 for each molecule across different gene set sizes.

SUPPLEMENTARY FIGURE S5 Distribution of BioPlanet gene sets containing BMD values which pass Filter F3 for the tested molecules: **(A)**: experiment-independent distribution of gene sets based on their sizes, serving as a reference to interpret the other three experiment-specific plots; **(B)**: distribution of gene sets passing Filter F3 for all molecules combined; **(C)**: number of gene sets passing Filter F3 for each molecule across different gene set sizes; **(D)**: percentage of gene sets passing Filter F3 for each molecule across different gene set sizes.

SUPPLEMENTARY FIGURE S6 Distribution of REACTOME gene sets containing BMD values which pass Filter F3 for the tested molecules: **(A)**: experiment-independent distribution of gene sets based on their sizes, serving as a reference to interpret the other three experiment-specific plots; **(B)**: distribution of gene sets passing Filter F3 for all molecules combined; **(C)**: number of gene sets passing Filter F3 for each molecule across different gene set sizes; **(D)**: percentage of gene sets passing Filter F3 for each molecule across different gene set sizes.

SUPPLEMENTARY FIGURE S7 Distribution of WGCNA gene sets containing BMD values which pass Filter F3 for the tested molecules: **(A)**: experiment-independent distribution of gene sets based on their sizes, serving as a reference to interpret the other three experiment-specific plots; **(B)**: distribution of gene sets passing Filter F3 for all molecules combined; **(C)**: number of gene sets passing Filter F3 for each molecule across different gene set sizes; **(D)**: percentage of gene sets passing Filter F3 for each molecule across different gene set sizes.

SUPPLEMENTARY TABLE S1 Variations of the gene set enrichment filters tested.

SUPPLEMENTARY DATA SHEET S1 Normalized and log-transformed (base 2) microarray gene expression data for the 79 TG-Gates molecules tested in this study.

SUPPLEMENTARY DATA SHEET S2 Annotation files for the four gene set structures used in the experiments (GOBP, BioPlanet, REACTOME, WGCNA).

SUPPLEMENTARY DATA SHEET S3 List of probesets used in Scenario 3 of the gene set enrichment from artificially down-sampled gene expression data.

SUPPLEMENTARY DATA SHEET S4 Concordance results for all comparisons discussed in the manuscript.

SUPPLEMENTARY DATA SHEET S5 Transcriptomic POD values for all gene set structures across 11 different gene set enrichment filters.

SUPPLEMENTARY DATA SHEET S6 Results for randomly-generated gene sets (1000 simulations) based on the structure of GOBP—allowing overlapping random gene sets.

SUPPLEMENTARY DATA SHEET S7 Results for randomly-generated gene sets (1000 simulations) based on the structure of BioPlanet—allowing overlapping random gene sets.

SUPPLEMENTARY DATA SHEET S8 Results for randomly-generated gene sets (1000 simulations) based on the structure of REACTOME—allowing overlapping random gene sets.

SUPPLEMENTARY DATA SHEET S9 Results for randomly-generated gene sets (1000 simulations) based on the structure of WGCNA—allowing overlapping random gene sets.

SUPPLEMENTARY DATA SHEET S10 Results for randomly-generated gene sets based on the structure of WGCNA enforcing non-overlapping random gene sets.

SUPPLEMENTARY DATA SHEET S11 Median tPOD across all simulations for the results from randomly-generated gene sets included in Supplementary Data Sheets S6–S10.

SUPPLEMENTARY DATA SHEET S12 Results for the gene set-based methods using reduced gene expression data (1000 simulations) from three different downsampling scenarios.

SUPPLEMENTARY DATA SHEET S13 Median tPOD across all simulations for the results from the reduced gene expression data included in Supplementary Data Sheet S12 (gene set-based methods).

SUPPLEMENTARY DATA SHEET S14 Distribution-based tPOD results for the original datasets.

SUPPLEMENTARY DATA SHEET S15 Results for the distribution-based methods using reduced gene expression data (1000 simulations) from three different downsampling scenarios.

SUPPLEMENTARY DATA SHEET S16 Median tPOD across all simulations for the results from the reduced gene expression data included in Supplementary Data Sheet S15 (distribution-based methods).
