# Supplementary figures and images for: Transcriptomic point of departure determination: a comparison of distribution-based and gene set-based approaches
Source: Front Genet. 2024 May 9;15:1374791. doi: 10.3389/fgene.2024.1374791 (PMC11112360; doi:10.3389/fgene.2024.1374791)

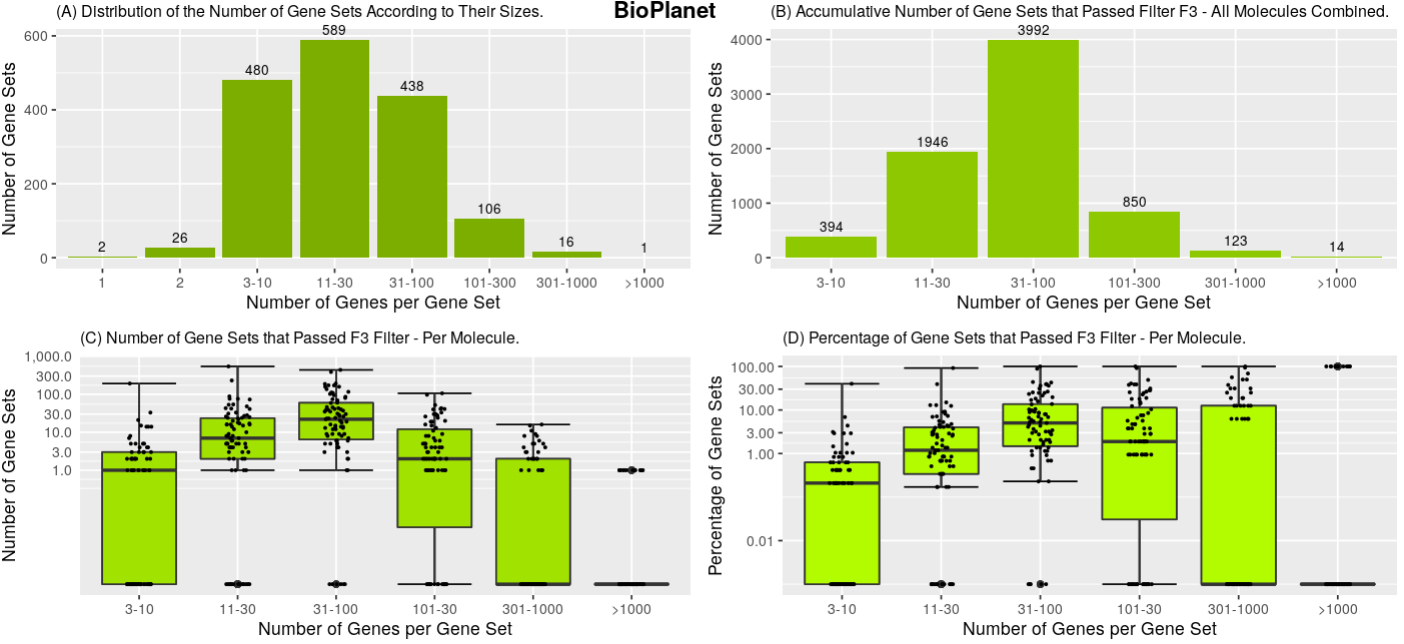

Supplement: Supplementary file 1 [file Image5.jpg]

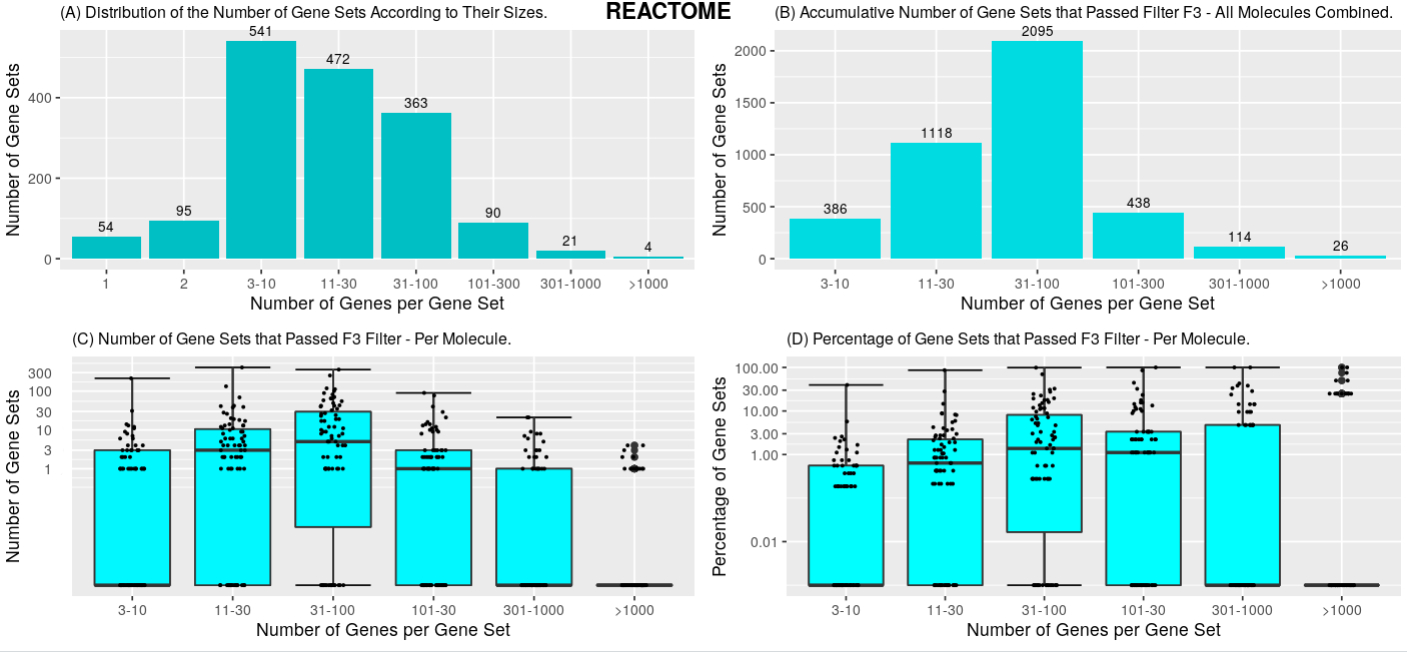

Supplement: Supplementary file 3 [file Image6.jpg]

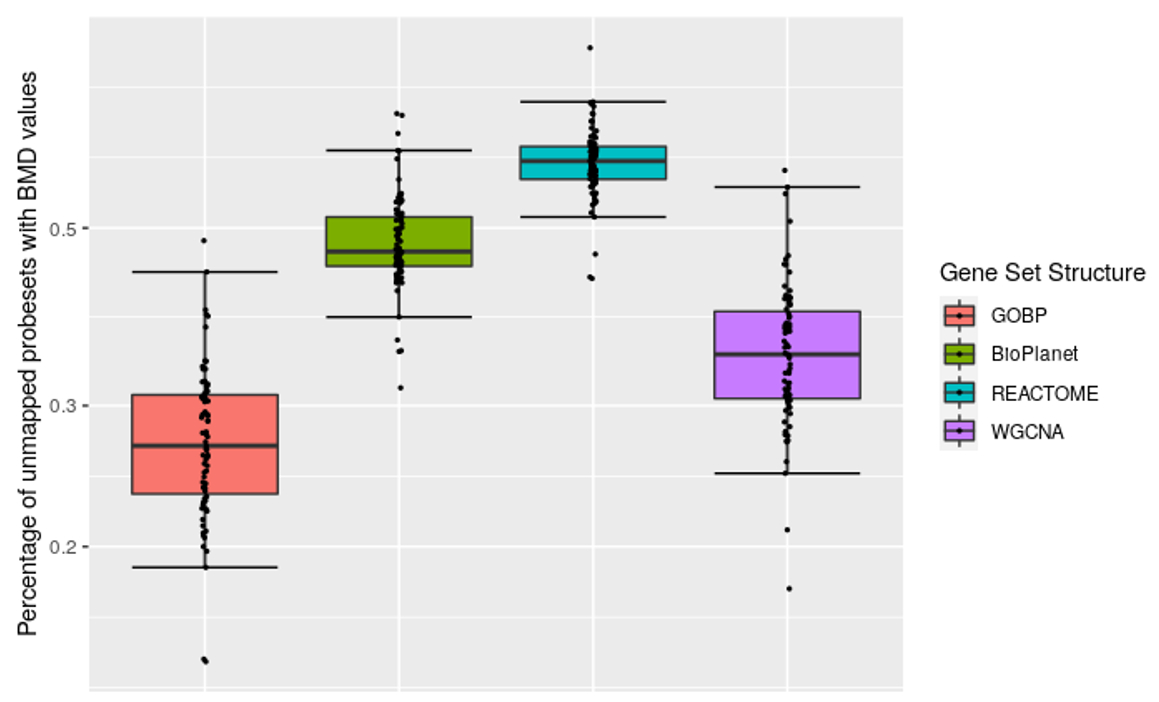

Supplement: Supplementary file 4 [file Image3.jpg]

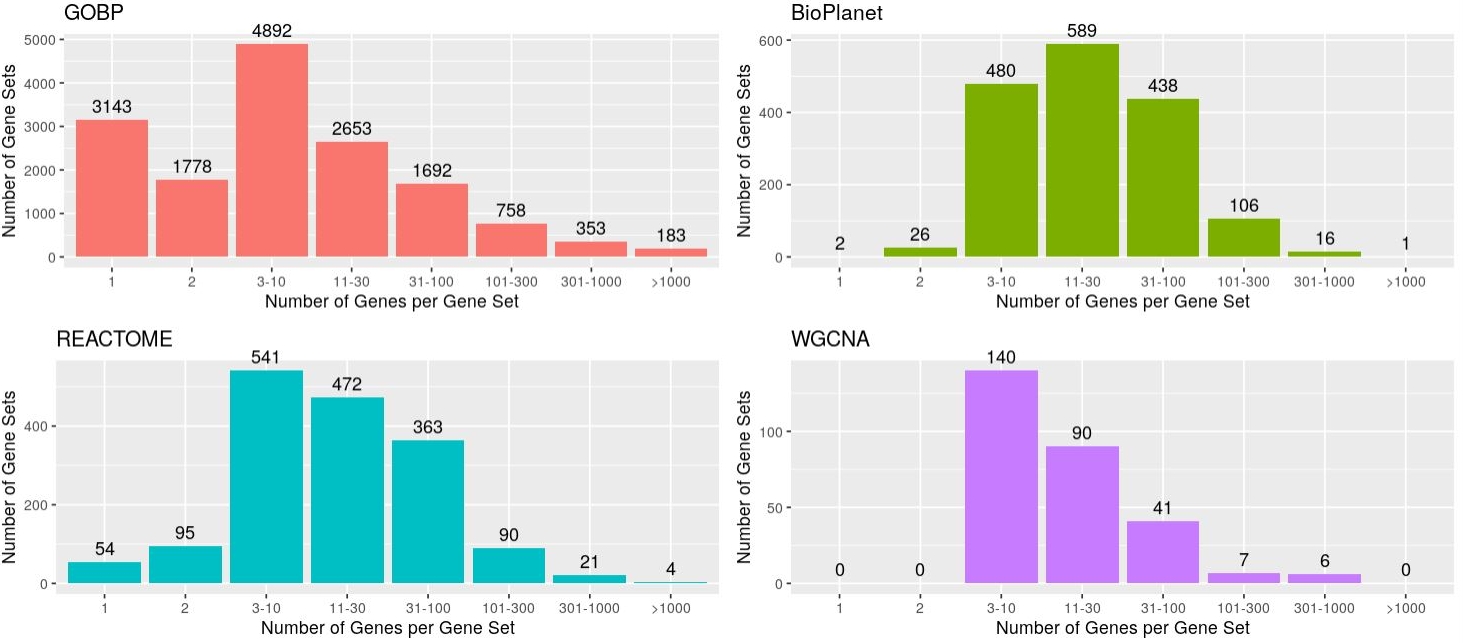

Supplement: Supplementary file 5 [file Image2.jpg]

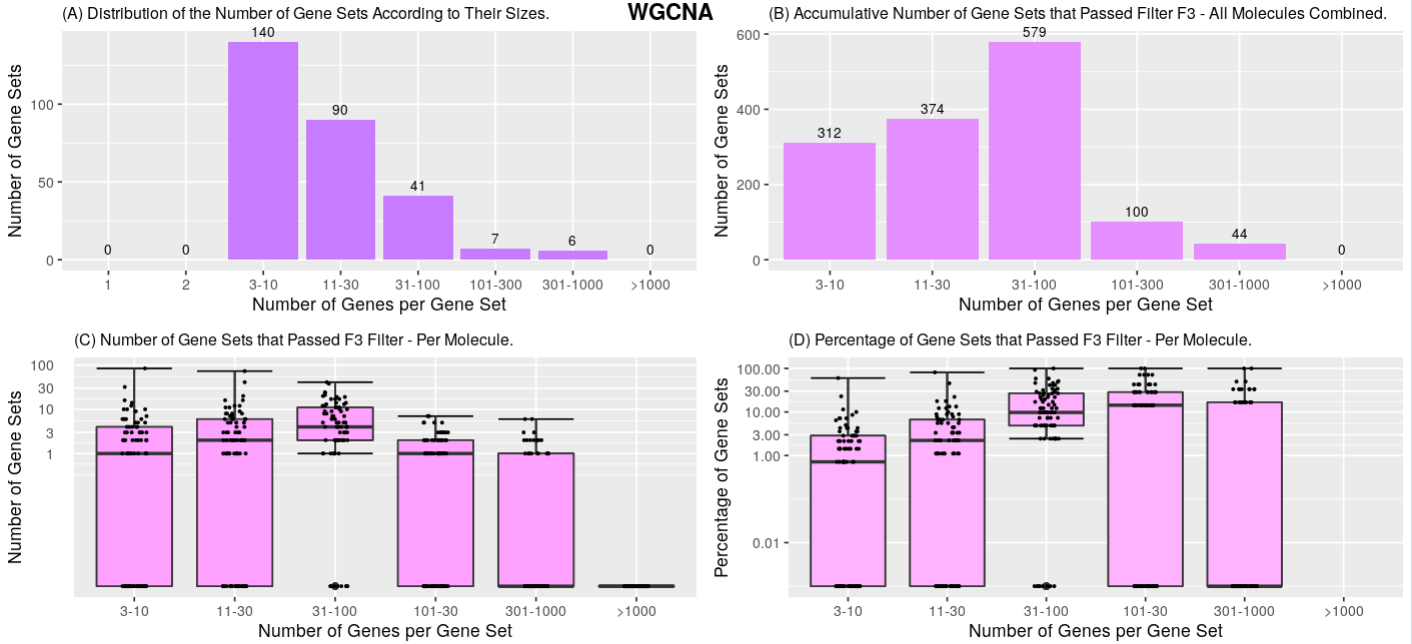

Supplement: Supplementary file 8 [file Image7.jpg]

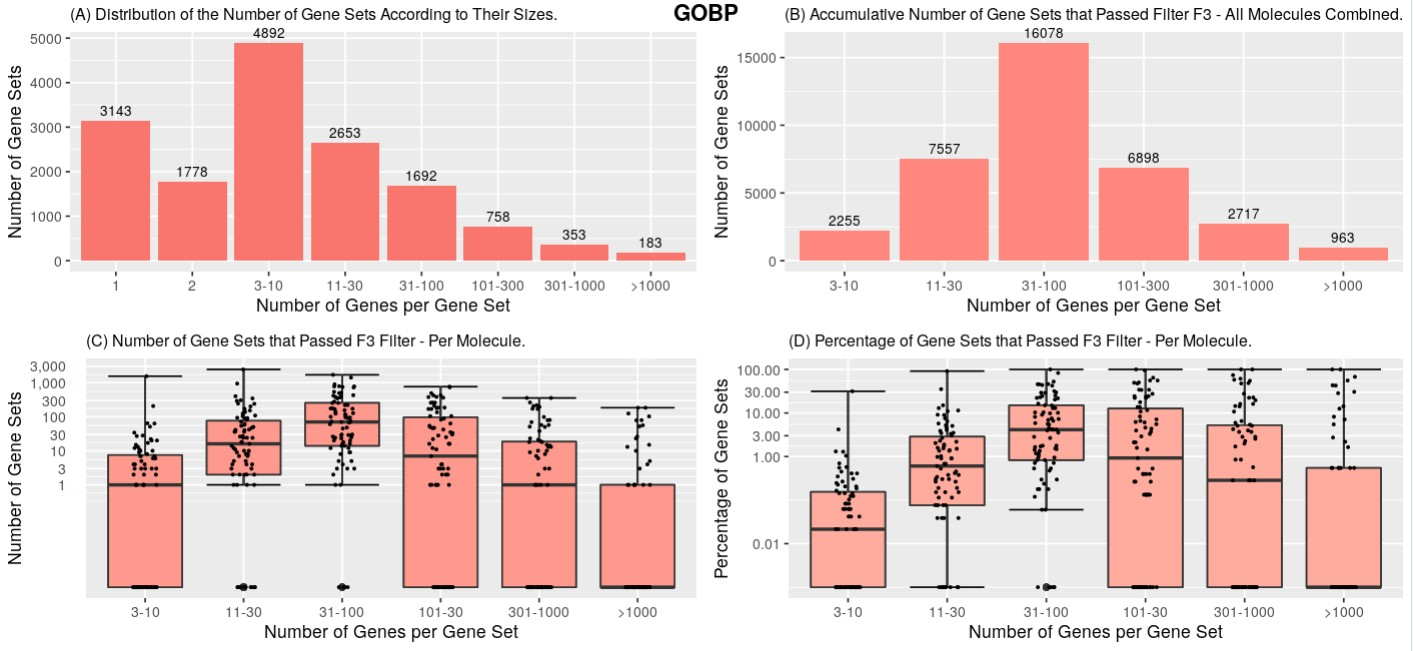

Supplement: Supplementary file 10 [file Image4.jpg]

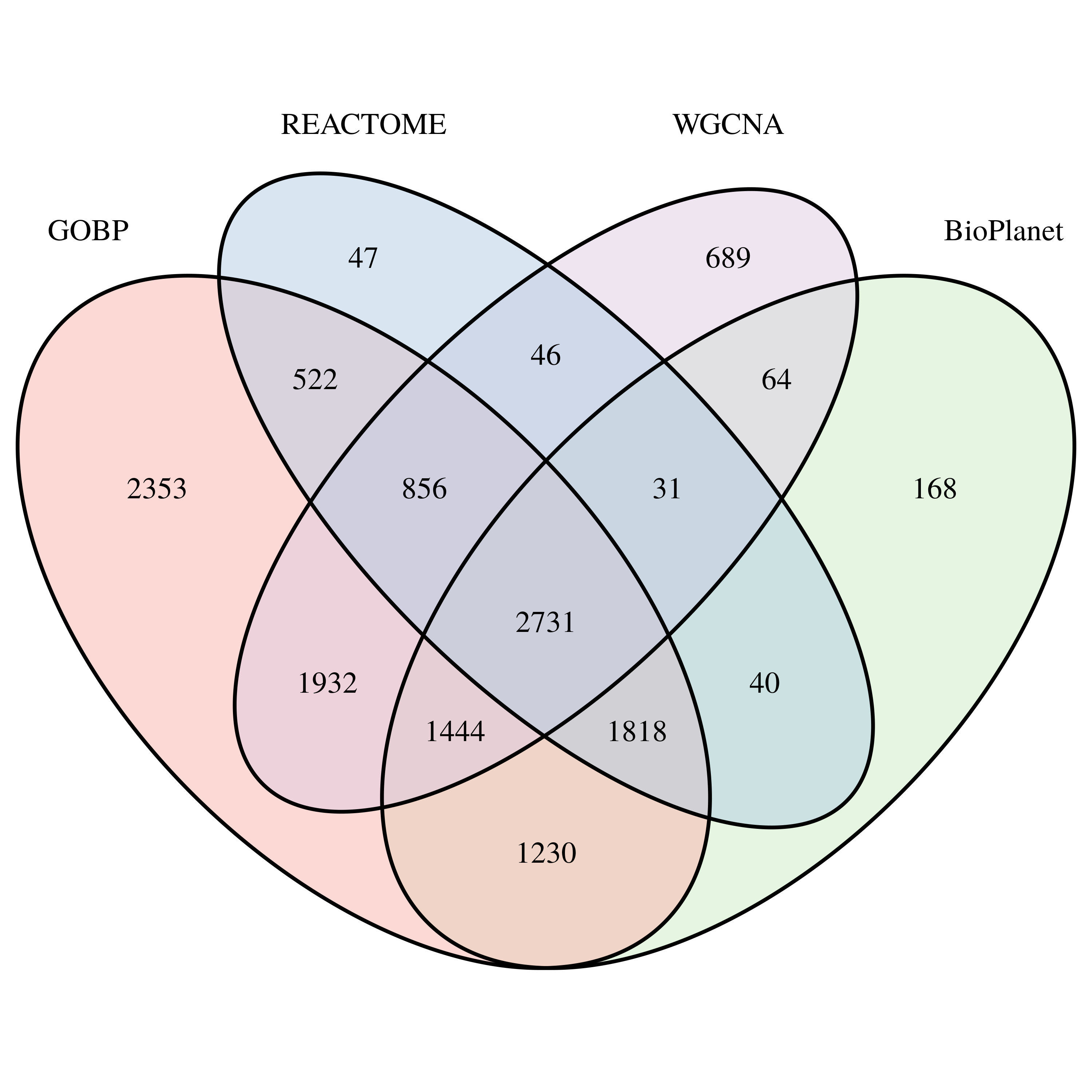

Supplement: Supplementary file 11 [file Image1.jpg]
